# Targeted Alpha Therapy: Exploring the Clinical Insights into [225Ac]Ac-PSMA and Its Relevance Compared with [177Lu]Lu-PSMA in Advanced Prostate Cancer Management

**DOI:** 10.3390/ph18081215

**Published:** 2025-08-18

**Authors:** Wael Jalloul, Vlad Ghizdovat, Tamás Pócza, Alexandra Saviuc, Despina Jalloul, Irena Cristina Grierosu, Cipriana Stefanescu

**Affiliations:** 1 Department of Biophysics and Medical Physics-Nuclear Medicine, “Grigore T. Popa” University of Medicine and Pharmacy, 700115 Iasi, Romania; jalloul.wael@umfiasi.ro (W.J.); iuliana.saviuc@gmail.com (A.S.); despina.prisacariu.dp@gmail.com (D.J.); irena.raileanu@umfiasi.ro (I.C.G.); cipriana.stefanescu@umfiasi.ro (C.S.); 2 North East Regional Innovative Cluster for Structural and Molecular Imaging (Imago-Mol), 700115 Iasi, Romania; 3 Institute of Nuclear Techniques, Budapest University of Technology and Economics, H-1111 Budapest, Hungary; poczatamas87@gmail.com; 4 Center of Radiotherapy, National Institute of Oncology, H-1122 Budapest, Hungary

**Keywords:** targeted alpha therapy, actinium-225, PSMA, radioligand therapy, metastatic castration-resistant prostate cancer, adverse effects, lutetium-177, [68Ga]Ga-PSMA PET/CT, theranostics

## Abstract

Targeted alpha therapy (TAT) has recently emerged as a highly promising approach for the management of metastatic castration-resistant prostate cancer (mCRPC), especially in patients with disease progression despite standard treatments. Among alpha-emitter radiopharmaceuticals, actinium-225-labelled prostate-specific membrane antigen ([225Ac]Ac-PSMA) has shown remarkable potential due to its high linear energy transfer (LET), short path length, and ability to induce potent, localised cytotoxic effects. This review summarises current clinical evidence regarding [225Ac]Ac-PSMA radioligand therapy (RLT), emphasising its efficacy, safety profile, and position relative to beta-emitter therapy with lutetium-177 ([177Lu]Lu-PSMA). Data from compassionate-use programs and small clinical trials demonstrate that [225Ac]Ac-PSMA produces significant biochemical and imaging responses, including > 50% declines in prostate-specific antigen (PSA) and lesion regression on [68Ga]Ga-PSMA PET/CT, even in heavily pre-treated mCRPC cohorts. Xerostomia, renal toxicity, and haematological adverse effects remain the main safety challenges, necessitating optimisation of patient selection, dosing strategies, and salivary gland protection protocols. Compared with [177Lu]Lu-PSMA, [225Ac]Ac-PSMA appears effective even in cases of beta-refractory disease, highlighting its complementary role rather than a competitive alternative. However, limited availability, high production costs, and the lack of large-scale, randomised trials hinder widespread clinical adoption. Future directions include combination protocols, improved radiopharmaceutical design, and trials evaluating its use in earlier disease stages. This review provides a comprehensive overview of the clinical aspects of [225Ac]Ac-PSMA RLT and its evolving role in advanced prostate cancer management.

## 1. Introduction

Existing genomic and proteomic research has provided important insights into the biological effects of TAT at the cellular and molecular levels. Studies have reported differential gene expression patterns, DNA repair pathway activation, and changes in protein profiles following both beta- and alpha-emitter exposure, highlighting potential biomarkers of response and resistance. While these findings contribute to understanding the mechanisms of action and may guide future personalised treatment strategies, they remain primarily in the preclinical stage. This review focuses on clinical data, practical treatment protocols, safety management, and the evolving prospective trial landscape. This targeted approach aims to provide clinicians and nuclear medicine specialists with a concise and up-to-date synthesis of evidence relevant to real-world practice.

Prostate cancer is the most frequently diagnosed malignancy and the second leading cause of cancer-related deaths among men in Europe [1,2]. Despite initial responsiveness to androgen deprivation therapy (ADT), many patients eventually develop mCRPC, which is associated with poor prognosis and limited treatment options [3]. Innovative treatment strategies are thus urgently required to extend survival and improve quality of life in this patient population.

RLT targeting PSMA has emerged as a novel and promising treatment modality. PSMA is a transmembrane protein with significantly increased expression in prostate cancer cells, particularly in the castration-resistant phase [4,5]. The therapeutic concept involves coupling a radionuclide to a small-molecule ligand with high affinity for PSMA, thereby delivering localised radiation to tumour cells while minimising exposure to normal tissues.

While β-emitting agents, such as Lu-177, have already entered clinical practice, alpha-emitting radionuclides, such as Ac-225, offer distinct radiobiological advantages. [225Ac]Ac-PSMA is rapidly gaining recognition as one of the most promising radiopharmaceuticals for TAT, especially in managing mCRPC. This novel therapeutic agent combines Ac-225, a potent alpha-emitting radioisotope, with a PSMA ligand [6]. The use of alpha particles offers several distinct advantages over beta-emitting therapies, including a high LET and short path length, allowing for the delivery of highly cytotoxic radiation to tumour cells while sparing nearby healthy tissue [7,8,9].

In studies on [225Ac]Ac-PSMA RLT, the most widely used PSMA-targeting ligands are PSMA-617 and PSMA I&T. These two molecules share the same PSMA-binding motif (Glu-urea-Lys), but differ structurally in their linkers and chelators. PSMA-617 incorporates a DOTA chelator, whereas PSMA I&T uses a DOTAGA chelator, which results in slightly different pharmacokinetic and dosimetry characteristics. As this review focuses on the general clinical aspects of [225Ac]Ac-PSMA RLT rather than a head-to-head comparison of Ac-225-PSMA-617 versus Ac-225-PSMA I&T, differences between these two ligands are only briefly outlined here. Moreover, in the present paper, nearly all of the cited studies investigating [225Ac]Ac-PSMA RLT used the PSMA-617 ligand.

## 2. Mechanism of Action of [225Ac]Ac-PSMA RLT

[225Ac]Ac-PSMA RLT is produced to deliver highly targeted alpha radiation to prostate cancer cells by exploiting the overexpression of PSMA. The therapeutic mechanism comprises four key components:

### 2.1. PSMA Targeting and Internalisation

PSMA is a type II transmembrane glycoprotein with glutamate carboxypeptidase activity, significantly upregulated in mCRPC [10]. The PSMA-617 ligand exhibits high-affinity (Ki ≈ 0.37 nM) binding to PSMA, triggering rapid receptor-mediated endocytosis via clathrin-coated pits [11]. This results in efficient internalisation of the radioligand and prolonged intracellular retention, both critical for effective cytotoxic delivery [12,13].

### 2.2. Alpha-Particle Emission and DNA Damage

Ac-225 emits four α-particles per decay chain, each delivering high linear energy transfer (LET ~100 keV/µm) over a short range (~ 47–85 µm), equivalent to just a few cell diameters [14,15]. This radiation induces dense clusters of DNA double-strand breaks, which are biologically more lethal and less repairable than those caused by β-particle radiation [14,15].

### 2.3. Dosimetric Advantage in Micrometastatic Disease

Preclinical dosimetry analyses have shown that [225Ac]Ac-PSMA delivers substantially higher radiation doses per decay than [177Lu]Lu-PSMA. When accounting for a relative biological effectiveness (RBE) of five, the absorbed dose is estimated to be approximately 2320 times greater in single cells, 2900 times in micrometastases with a 100 µm radius, and around 823 times in macroscopic tumours [16]. Precisely targeted radiation pattern minimises cross-fire, making Ac-225 particularly effective against small tumour foci such as micrometastases and circulating tumour cells.

### 2.4. Chelation Stability and Biodistribution

PSMA-617, for example, employs a DOTA chelator to stably bind Ac-225, retaining the radionuclide and its daughter products within tumour cells [17]. This reduces off-target dissociation, minimising radiation to non-target organs. The resulting biodistribution is characterised by deep tumour penetration, rapid clearance from non-target tissues, and reduced systemic toxicity [18,19].

**Figure 1:**
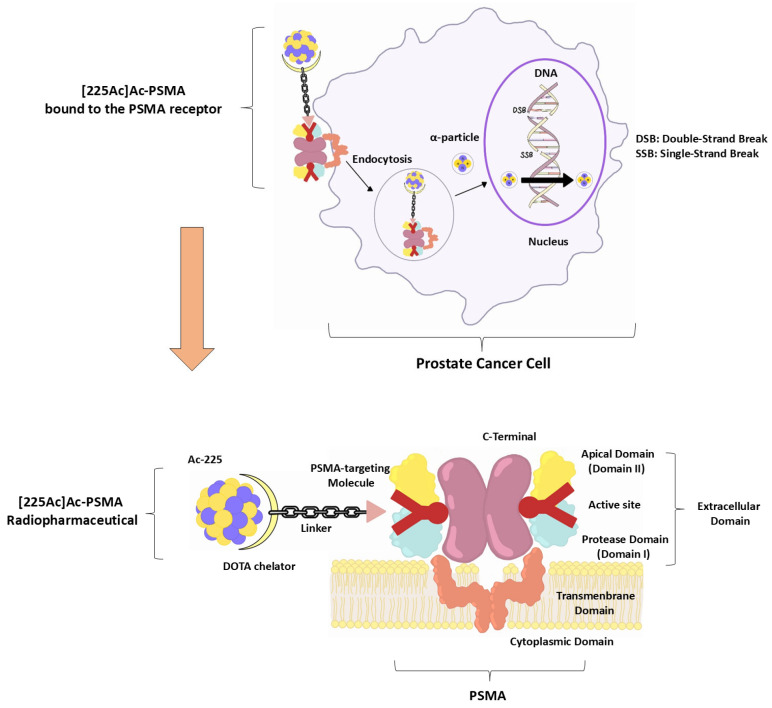
Mechanism of action of [225Ac]Ac-PSMA RLT.

In essence, [225Ac]Ac-PSMA combines high-affinity PSMA targeting, potent cellular internalisation, and highly localised alpha radiation to achieve targeted tumour cytotoxicity. Its radiobiological profile addresses limitations of traditional β-emitters, particularly for micrometastatic disease, and offers the potential for durable tumour control in PSMA-positive prostate cancer (Figure 1) [20,21,22].

## 3. Clinical Indications and Patient Selection

The current clinical use of [225Ac]Ac-PSMA RLT is focused on patients with mCRPC who have experienced disease progression following standard systemic therapies. Selection criteria are guided by evidence from phase I/II clinical trials and large multicentre retrospective cohorts, with particular emphasis on PSMA expression, tumour burden, performance status, and prior treatment history.

PSMA expression, assessed by PET/CT imaging, is essential for patient eligibility in PSMA RLT. A sufficient expression is typically defined as radiotracer uptake in metastatic lesions that exceeds the physiologic background uptake of the normal liver [23]. Some studies further refine this selection by requiring a tumour-to-liver SUV ratio > 1.0 and SUVₘₐₓ > 15 with homogeneous uptake across lesions [24]. These imaging-based thresholds help ensure adequate target density necessary to justify alpha-emitter exposure and optimise tumour cell uptake [25].

Patients eligible for [225Ac]Ac-PSMA therapy typically exhibit an Eastern Cooperative Oncology Group (ECOG) performance status of ≤ 2, castrate-level serum testosterone concentrations (<1.7 nmol/L), and evidence of progressive disease. Disease progression is defined by rising PSA levels, the appearance of new bone lesions, or measurable progression on imaging, as per RECIST 1.1 or Prostate Cancer Clinical Trials Working Group 3 (PCWG3) criteria. Additional laboratory thresholds include haemoglobin ≥ 9 g/dL, absolute neutrophil count ≥ 1.5 × 10^9^/L, platelets ≥ 100 × 10^9^/L, creatinine clearance ≥ 50 mL/min, and liver enzymes within twice the upper limit of normal [26,27,28].

Clinical data from the Actinium225PSMA Radioligand Therapy of Metastatic Castration-Resistant Prostate Cancer (WARMTH Act) study, which is a large multicentre cohort involving 488 patients from seven international centres, confirmed that [225Ac]Ac-PSMA RLT is most often used in heavily pre-treated mCRPC patients. In this cohort, 66.2% had previously received docetaxel, 21.3% cabazitaxel, 38.8% abiraterone, 38.5% enzalutamide, and 32% had progressed on prior [177Lu]Lu-PSMA RLT [29]. These findings support the current role of [225Ac]Ac-PSMA therapy as a late-line salvage option, especially after failure of both androgen receptor signalling inhibitors and beta-emitting RLT.

However, newer studies are expanding the therapeutic scope of [225Ac]Ac-PSMA RLT. In a prospective study by M. Sathekge et al. (2022) [30], patients with mCRPC treated with [225Ac]Ac-PSMA after ADT, but before chemotherapy or [177Lu]Lu-PSMA, achieved > 50% decline (PSA50) response in 91% of cases. Furthermore, in a prospective study involving hormone-sensitive metastatic prostate cancer (mHSPC), M. Sathekge et al. (2023) [31] reported that 19% of patients achieved undetectable PSA levels following treatment with [225Ac]Ac-PSMA-617, with median progression-free survival (PFS) exceeding 12 months.

Ongoing trials such as AcTION (NCT04597411) and SatisfACTion (NCT05983198) apply strict inclusion criteria consistent with the above, while also investigating the role of [225Ac]Ac-PSMA in patients with low-volume disease, oligometastatic states, and post-[177Lu]Lu-PSMA RLT [26,32].

At present, the primary indication for [225Ac]Ac-PSMA remains mCRPC in patients who have exhausted standard-of-care therapies. However, emerging evidence suggests significant efficacy when used earlier in the treatment algorithm, including in hormone-sensitive and [177Lu]Lu-PSMA-resistant disease. Careful patient selection based on PSMA expression, prior treatment, and organ function is critical to optimising therapeutic outcomes and minimising toxicity.

### 3.1. Eligibility Criteria Typically Include the Following:

❖ Histologically confirmed mCRPC.
❖ Progressive disease despite androgen receptor signalling inhibitors (e.g., enzalutamide, abiraterone) and/or taxane-based chemotherapy.
❖ Adequate organ function (e.g., bone marrow, renal, hepatic reserve).
❖ PSMA expression on PET/CT using [68Ga]Ga-PSMA or [18F]F-PSMA ligands (i.e., tumour uptake ≥ liver background).
❖ ECOG performance status ≤ 2.

### 3.2. Exclusion Criteria Often Include the Following:

❖ Diffuse bone marrow infiltration.
❖ Poor PSMA expression on imaging.
❖ Prior to myelotoxic therapy <6 weeks before RLT.
❖ Active uncontrolled infections or autoimmune diseases.

## 4. Administration Protocols and Dosimetry

The dosing and administration schedules of [225Ac]Ac-PSMA exhibit considerable variability across clinical trials, indicating a field that remains under active optimisation (Table 1). 

Both fixed-dosing and weight-based strategies have been employed in an attempt to balance efficacy with toxicity. In several prospective studies, such as those conducted by Sathekge and colleagues (2019; 2020) [8,34], treatment was initiated with a fixed dose of 8 MBq per cycle. Depending on patient response and tolerability, this dose was subsequently reduced to 7, 6, or even 4 MBq. Most patients in these studies received three cycles, administered every eight weeks, though therapy was occasionally halted earlier in cases of rapid clinical improvement or extended for up to eight cycles when needed. In one notable case, a dose escalation to 13 MBq was applied during the third cycle in response to suboptimal tumour control.

Weight-based dosing has also been adopted as a strategy for personalising therapy, particularly using a standardised activity of 100 kBq/kg body weight per cycle. Yadav et al. (2020) [7] administered two such cycles at eight-week intervals, assessing patients at six weeks post-treatment to determine whether therapy should be discontinued due to disease progression or continued in the event of stable or responsive disease. A similar dosing protocol was used in the prospective study by Kratochwil et al. (2018) [9], where patients received between three and five cycles. Retrospective studies have reinforced the consistency of this approach, with Sen et al. (2021) [38] and Satapathy et al. (2020) [28] both employing median doses of approximately 100 kBq/kg across two to four cycles, spaced between eight and twelve weeks apart.

Beyond these standardised approaches, data reveal a broader range of dosing strategies. In a retrospective pooled analysis encompassing 263 patients across nine studies, Lee and Kim (2022) [33] reported doses ranging from 1.5 to 13 MBq per cycle, with the number of administered cycles varying between one and eight. These deviations reflect pragmatic adjustments based on patient tolerance, tumour burden, and prior treatment exposure. Other retrospective series, such as those by Rosar et al. (2021) [37] and Feuerecker et al. (2021) [36], indicated average administered doses between 2.7 and 9 MBq per cycle, with most patients undergoing one to six treatment sessions.

Of particular interest are studies exploring tandem therapy approaches, combining [225Ac]Ac-PSMA with [177Lu]Lu-PSMA. Khreish et al. (2020) [39], for example, administered a median of 5.3 MBq of [225Ac]Ac-PSMA in conjunction with 6.9 GBq of [177Lu]Lu-PSMA in a single treatment cycle. Responders to this initial combination were subsequently maintained on [177Lu]Lu-PSMA monotherapy for up to five additional cycles, highlighting a flexible approach aimed at overcoming resistance following monotherapy failure.

Across studies, the most common inter-cycle interval was eight weeks, although flexibility was observed depending on clinical response and toxicity. In some cases, such as in Satapathy et al. (2020) [28], treatment intervals were extended to up to twelve weeks. The number of cycles administered was also variable, with a median of two to three cycles being typical, although a small proportion of patients received up to eight cycles when sustained disease control was achieved.

Overall, the administration of [225Ac]Ac-PSMA remains heterogeneous, shaped by ongoing dose optimisation studies, patient-specific considerations, and resource availability. The consistent use of eight-week dosing intervals and body-weight-adjusted activity at 100 kBq/kg has emerged as a provisional standard, though flexible dose reduction based on toxicity remains critical. As clinical experience continues to expand and more robust prospective data become available, dosing protocols are likely to become increasingly refined and individualised. This therapeutic variability underscores the growing role of personalised medicine in [225Ac]Ac-PSMA treatment, where dosing strategies and cycle schedules are increasingly being adapted to each patient’s biological profile, tumour PSMA expression levels, prior treatment history, and early response indicators such as PSA kinetics or molecular imaging. For instance, patients with high tumour burden and low baseline haematological reserve may benefit from initial dose de-escalation, while those with aggressive or radioresistant disease might require intensification or tandem approaches. Furthermore, quantitative PSMA PET/CT imaging is emerging as an essential tool for pre-therapeutic assessment, allowing for stratification and individualisation of both activity and cycle frequency. These developments reflect a shift away from one-size-fits-all protocols toward more nuanced, patient-specific management in the era of targeted alpha therapy.

Another noteworthy point is that the optimal sequencing of [225Ac]Ac-PSMA within the mCRPC treatment algorithm may influence both efficacy and tolerability. Most retrospective and prospective cohorts to date have positioned [225Ac]Ac-PSMA RTL as a late-line salvage option, administered after progression on androgen receptor signalling inhibitors, taxane-based chemotherapy, and/or [177Lu]Lu-PSMA RTL [7,8,28,29,34,38]. However, prospective research in chemotherapy-naïve patients [8,30] and even in hormone-sensitive metastatic settings [31] has reported high PSA response rates (often > 80% PSA50) with manageable toxicity, suggesting that earlier integration may allow for lower cumulative doses while maintaining durable disease control. These observations point toward a model in which both dose and treatment position within the sequence are tailored to individual patient profiles. The influence of sequencing on long-term outcomes is now being formally investigated in ongoing trials such as AcTION (NCT04597411) and SatisfACTion (NCT05983198), which are expected to inform standardised algorithms for both activity selection and treatment timing.

## 5. Response Evaluation and Outcomes

Evaluation of therapeutic response following [225Ac]Ac-PSMA RLT in mCRPC involves a multimodal assessment, including both biochemical and imaging criteria, alongside clinical parameters.

### 5.1. Biochemical Response—PSA

Serum PSA levels remain the most frequently used biomarker. A ≥50% PSA decline from baseline is typically considered a favourable response [40,41]. However, sole reliance on PSA is limited by the occurrence of PSA flare phenomena, discrepancies between biochemical and radiological responses, and a lack of correlation in cases with neuroendocrine differentiation or dedifferentiated tumour phenotypes [42,43,44].

### 5.2. Imaging-Based Assessment

Post-treatment imaging is most commonly performed using [68Ga]Ga-PSMA PET/CT, although [18F]F-PSMA PET/CT is also utilised. These modalities enable assessment of disease burden reduction, evaluation of concordance between imaging findings and PSA dynamics, and identification of new or progressing lesions.

Recent advances in quantitative PSMA PET parameters are increasingly adopted in research, including the following [45,46,47,48,49,50]:

❖ SUVₘₐₓ reduction: often used as a robust semiquantitative parameter with high reproducibility.
❖ Whole-body tumour volume (PSMA TV): the total volume of PSMA-avid lesions; higher baseline PSMA TV is a statistically significant negative predictor of overall and PFS.
❖ Total lesion PSMA (TL PSMA): the product of PSMA TV by SUVmean, analogous to total lesion glycolysis (TLG) in [18F]F-FDG PET; elevated TL PSMA correlates with worse prognosis.

SUVₘₐₓ, PSMA TV, and TL PSMA play a crucial role in standardising response evaluation beyond visual interpretation, enabling early detection of treatment failure, often before PSA rise is observed, and stratifying prognosis based on initial lesion burden and uptake.

In good responders to [225Ac]Ac-PSMA RLT, PET imaging may demonstrate one of the following patterns:

❖ Complete metabolic response: no detectable PSMA uptake.
❖ Partial response: decrease in the number or size of PSMA-avid lesions.
❖ Stable disease: no significant change in lesion burden; that is, neither new nor regressing lesions.

### 5.3. Survival Outcomes

Despite the limited number of prospective studies, survival outcomes with [225Ac]Ac-PSMA-617 are encouraging, particularly in heavily pre-treated populations.

In one of the earliest and most cited retrospective series, Yadav et al. (2020) [7] reported on a cohort of heavily pre-treated mCRPC patients who received [225Ac]Ac-PSMA. The study demonstrated a median PFS of 12 months and a median overall survival (OS) of 17 months. Notably, 63% of patients experienced a PSA decline of ≥50%, a response associated with improved survival metrics. These results were achieved in a population with limited treatment options, underlining the potential of alpha therapy even in advanced stages of disease.

Similar findings have been observed in additional studies. M. Sathekge et al. (2020) [8], in a South African cohort, reported a median PFS of 12 months in patients treated with [225Ac]Ac-PSMA after prior exposure to chemotherapy and/or [177Lu]Lu-PSMA. Importantly, patients with a greater PSMA uptake on baseline PET/CT scans and lower tumour burden achieved better survival outcomes, reinforcing the role of imaging biomarkers in prognostication and patient selection.

Prognostic factors repeatedly associated with improved outcomes across studies include high PSMA expression on PET, low PSMA-TV, fewer prior lines of systemic therapy, and preserved ECOG performance status [51]. These variables reflect both tumour biology and host condition, and have been proposed as part of a multimodal risk stratification framework for selecting patients most likely to benefit from alpha therapy.

Additionally, Gafita et al. (2022) [51] demonstrated that quantitative imaging biomarkers such as total lesion TL-PSMA and PSMA-TV were independently associated with OS and PFS in multivariable models. For each doubling of PSMA-TV, the hazard ratio for death increased by approximately 1.4, suggesting a meaningful impact of baseline disease burden on survival. These findings support the integration of volumetric imaging metrics into therapeutic decision-making and response monitoring.

Although randomised controlled trial data are still forthcoming, real-world evidence has laid the groundwork for defining realistic expectations. Median survival durations of 12–18 months in a salvage setting, especially for patients who had exhausted conventional therapies, position [225Ac]Ac-PSMA as a potent therapeutic option with curative potential in selected cases.

## 6. Side Effects and Toxicity

Xerostomia emerges as the most frequently reported adverse effect of [225Ac]Ac-PSMA RLT, attributable to the physiological expression of PSMA in salivary glands and the high energy deposition of alpha particles in small glandular tissue volumes (Table 2).

Multiple studies have confirmed its prevalence, with Sathekge et al. (2019) [8] observing Grade 1/2 xerostomia in 100% of patients. Similarly, meta-analyses by Ma et al. (2022) [53] and Ling et al. (2022) [54] reported incidence rates of 77.1% and 77%, respectively, for xerostomia of any grade, whereas Lee and Kim (2022) [33] reported a rate of 63%. Severe xerostomia (Grade ≥ 3), though less common, remains clinically significant, with Ma et al. (2022) [53] and Ling et al. (2022) [54] reporting rates of 3% and 17%, respectively. Alan-Selcuk et al. (2023) [55] also observed a 100% incidence of xerostomia, underscoring its universal occurrence across cohorts.

Haematologic toxicities, including anaemia, leukopenia, and thrombocytopenia, constitute the second major class of adverse events. Grade 3 anaemia was noted in 21.1% of patients by Perrone et al. (2025) [52] and in 5.9% by Sathekge et al. (2019) [8]. Meta-analytic findings indicate a consistent pattern: Ma et al. (2022) [53] reported anaemia in 30.3% of patients (Grade ≥ 3 in 7.5%), Lee and Kim (2022) [33] in 14%, and Ling et al. (2022) [54] in 31% (Grade ≥ 3 in 11%). Leukocytopenia occurred less frequently but remains a concern in heavily pre-treated populations; Grade 3 cases ranged from 4.5% [53] to 8.4% [52], with Lee and Kim (2022) [33] reporting 4%. Thrombocytopenia was observed in 14.9% of patients [53] and 15% [54], with Grade 3–4 events occurring in 5.5% to 19.7% across studies. Alan-Selcuk et al. (2023) [55] found haematologic toxicity of any grade in 17.4% of patients.

Renal toxicity, although less frequent than salivary or haematologic events, is a critical dose-limiting consideration, especially in patients with pre-existing renal impairment. Grade 3 nephrotoxicity was observed in 1.4% of patients by Perrone et al. (2025) [52] and in 5.9% by Sathekge et al. (2019), Ma et al. (2022), and Ling et al. (2022) [8,53,54] found Grade ≥ 3 nephrotoxicity rates of 3% and 7%, respectively, while any-grade renal toxicity was reported in 21% [54]. Alan-Selcuk et al. (2023) [55] reported nephrotoxicity (Grades 1–3) in 4.3% of patients, suggesting a consistent but moderate risk.

General systemic side effects such as fatigue, gastrointestinal discomfort, and constitutional symptoms are also commonly reported, albeit often underemphasised in early reports. Fatigue of any grade was present in 44–50% of patients [7,54], with severe fatigue (Grade ≥ 3) in 3.6–4%. Gastrointestinal adverse events, including anorexia (16.9–29%), nausea (12.9–21%), constipation (10.4–25%), and diarrhoea (9%), were variably reported across studies [53,54]. These effects, although mostly mild to moderate, can affect adherence and quality of life.

Additionally, less frequent but clinically noteworthy toxicities include hepatotoxicity and flare pain. Perrone et al. (2025) [52] documented Grade 3 hepatotoxicity in 8.4% of their cohort, and flare pain was reported in 22.5% of patients, potentially requiring analgesic management. Rare events such as xerophthalmia were observed in up to 6% of patients [53,54], and hearing loss was documented in 1%. Dysgeusia was reported by Ling et al. (2022) [54] in 11% of patients.

In summary, [225Ac]Ac-PSMA therapy is associated with a distinct toxicity profile dominated by xerostomia, followed by haematologic suppression and renal dysfunction. While most side effects are manageable, xerostomia remains dose-limiting in a substantial proportion of patients and demands preventive strategies. Haematologic and renal adverse events necessitate careful baseline assessment and monitoring, particularly in patients with prior extensive treatment histories. Ongoing protocol refinements, such as dose de-escalation and tandem therapy, aim to mitigate these toxicities while preserving efficacy.

### Mitigation Strategies and Long-Term Safety Considerations in Xerostomia

Several strategies have been explored to mitigate salivary gland toxicity, though none are yet standardised. Mechanical or physical interventions, such as the application of ice packs to the parotid and submandibular regions during radioligand infusion, aim to induce vasoconstriction and reduce glandular radiotracer uptake [8,39]. Pharmacological approaches, including the use of sialoprotective agents such as amifostine, pilocarpine, or botulinum toxin A injections into major salivary glands, have shown variable results in small patient series [8,53,54]. Pre-targeting strategies, ligand modification to reduce salivary gland affinity, and tandem therapy regimens (lowering per-cycle [225Ac]Ac-PSMA activity while combining with [177Lu]Lu-PSMA) are also under investigation as methods to limit cumulative glandular dose without compromising tumour control [39]. While some of these interventions have demonstrated symptomatic benefit, robust prospective data are lacking, and their efficacy in preventing irreversible xerostomia remains uncertain.

In terms of long-term safety, current evidence is limited by the relatively recent adoption of alpha-emitter PSMA therapies. Theoretical risks include therapy-related myelodysplastic syndromes (t-MDS) and secondary malignancies, potentially arising from cumulative bone marrow irradiation and off-target alpha particle deposition [8,53,54]. No definitive cases directly attributable to [225Ac]Ac-PSMA have been reported to date, but analogous experiences with other radiopharmaceuticals suggest that vigilance is warranted. Long-term follow-up protocols, such as periodic full blood counts, renal function testing, and clinical screening for late toxicities, are therefore recommended, particularly for younger patients or those achieving prolonged survival. Establishing prospective registries and extended follow-up within ongoing trials will be critical for defining the true incidence and spectrum of late adverse effects [26]. These frameworks should incorporate harmonised reporting standards, routine imaging and laboratory surveillance, and quality-of-life assessments to enable cross-centre comparability and pooled analyses [8,53,54]. Furthermore, linking long-term safety monitoring with studies evaluating integration of [225Ac]Ac-PSMA into multimodal regimens, such as tandem radioisotope therapy, androgen receptor pathway inhibition, or immunotherapy, may help identify both potential synergistic effects and cumulative toxicity patterns [39].

## 7. Comparison with [177Lu]Lu-PSMA

While both [225Ac]Ac-PSMA and [177Lu]Lu-PSMA target the same molecular structure, which is PSMA, they differ fundamentally in radiophysical properties, clinical efficacy, and toxicity profiles (Table 3) [14,17,56,57].

**Figure 2:**
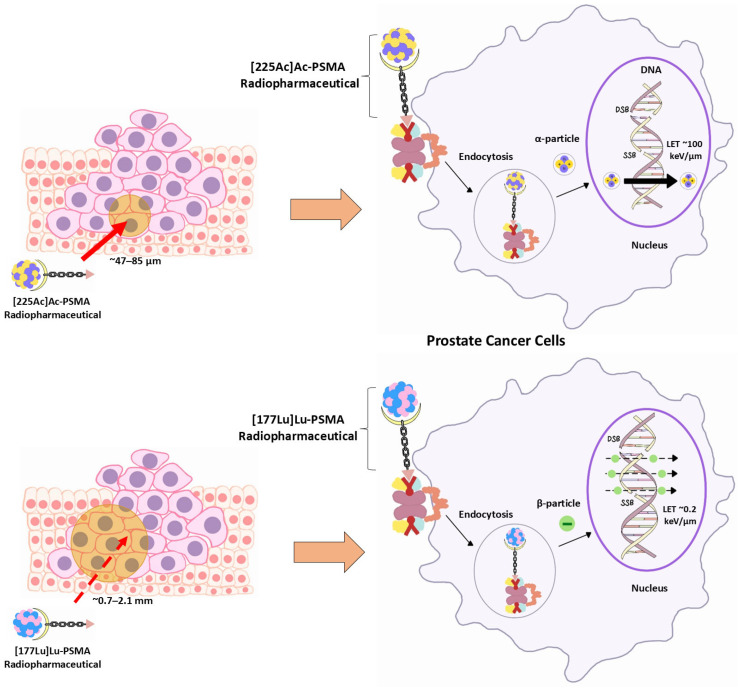
Relative biological effectiveness and DNA damage pathways of [225Ac]Ac-PSMA vs. [177Lu]Lu-PSMA.

Due to its short path length and high LET, Ac-225 delivers highly localised and lethal DNA damage, making it especially effective in micrometastatic or low-volume disease. In contrast, Lu-177’s longer path is more suited to larger tumour masses (Figure 2) [20,21].

### 7.1. Clinical Efficacy

Clinical evidence supporting the efficacy of [225Ac]Ac-PSMA RLT in advanced prostate cancer continues to accumulate, particularly in patients who are refractory to [177Lu]Lu-PSMA treatment. A retrospective analysis and systematic review by Stangl-Kremser et al. (2023) [58] reported that patients who exhibited disease progression following [177Lu]Lu-PSMA therapy showed renewed and, in some cases, pronounced biochemical and clinical responses after subsequent treatment with [225Ac]Ac-PSMA. These findings suggest a distinct therapeutic mechanism and potential for sequencing radionuclide therapies.

Despite encouraging outcomes from retrospective and non-randomised prospective studies, no head-to-head randomised clinical trials have yet directly compared [225Ac]Ac-PSMA with [177Lu]Lu-PSMA in equivalent patient cohorts. While retrospective series and small prospective studies suggest differing efficacy profiles, the lack of true randomised comparisons limits definitive conclusions [8,29,58]. Variations in patient populations, prior treatments, and imaging-based selection criteria further complicate indirect comparisons, rendering such analyses inherently uncertain. Therefore, cautious interpretation is essential, especially when extrapolating results to earlier treatment lines. Consequently, although preliminary efficacy data are promising, definitive conclusions regarding comparative superiority or optimal sequencing await confirmation through future prospective trials.

### 7.2. Dosimetry Considerations

Dosimetry for [225Ac]Ac-PSMA RLT remains significantly more complex than for [177Lu]Lu-PSMA, largely due to the absence of suitable gamma emissions from Ac-225 and its daughter isotopes for routine quantitative imaging. The short path length and highly potent LET of alpha particles also complicate spatial dose estimation. While [177Lu]Lu-PSMA allows patient-specific dosimetry via SPECT/CT, [225Ac]Ac-PSMA relies on surrogate approaches, such as pre- and post-treatment PET imaging using [68Ga]Ga-PSMA, combined with organ function monitoring and haematological parameters [59,60].

Recent advances, however, have enabled low-count quantitative SPECT imaging using the 440 keV gamma emission of Bismuth-213 (Bi-213), a daughter nuclide of Ac-225. This approach permits individualised dosimetry in clinical settings and is now under early clinical validation [15,59].

Radiobiologically, α-particle emitters such as Ac-225 exhibit a markedly higher RBE, often assumed to be around five times that of β-emitters such as Lu-177. As a result, therapeutic effects can be achieved at much lower administered activities. For example, approximately 100 kBq of [225Ac]Ac-PSMA is estimated to yield a tumouricidal impact comparable to 7.4 GBq of [177Lu]Lu-PSMA [16]. Subcellular dosimetry simulations further suggest that Ac-225 delivers up to 3000–4000 times more effective dose in micrometastatic disease and single cells, underscoring its potency in eradicating minimal residual disease [16].

### 7.3. Toxicity Profile

Compared with [177Lu]Lu-PSMA, [225Ac]Ac-PSMA therapy shows a higher incidence of xerostomia, while haematologic and renal toxicities are generally of similar magnitude (Table 4). These differences reflect the distinct biodistribution and radiobiological properties of alpha versus beta emitters, as previously detailed above. Long-term safety data for [225Ac]Ac-PSMA are still emerging, whereas [177Lu]Lu-PSMA has a more established and predictable toxicity profile. Careful patient selection and monitoring are essential to optimise safety outcomes with either agent [52,61,62,63,64].

### 7.4. Tandem and Sequential Strategies

Current evidence supports a complementary rather than competitive positioning of [225Ac]Ac-PSMA and [177Lu]Lu-PSMA [8,29,39,58,65]. Several clinical centres have investigated tandem regimens, which consist of administering both [177Lu]Lu-PSMA and [225Ac]Ac-PSMA either during the same treatment cycle or in close sequence. This therapeutic approach aims to harness the complementary properties of both radionuclides, utilising the deeper tissue penetration of Lu-177 to target voluminous tumour masses, while capitalising on the high LET and short path length of Ac-225 to eliminate micrometastatic disease and residual tumour cells.

In a pilot retrospective study involving 20 mCRPC patients who had insufficient response to [177Lu]Lu-PSMA monotherapy, tandem therapy was administered, consisting of a median of ~5.3 MBq [225Ac]Ac-PSMA and ~6.9 GBq [177Lu]Lu-PSMA per patient in the same cycle (typically consecutive days) [39]. The results were encouraging, with 65% of patients experiencing a PSA decline of > 50%, with alongside a median PFS of 19 weeks and OS of 48 weeks. Importantly, xerostomia was predominantly mild, with no Grade 3 or 4 cases, suggesting a favourable toxicity profile under this regimen [39]. Moreover, recent original studies and systematic review/meta-analysis reported similar tandem approaches and emphasised that combining Lu-177 and Ac-225 may enhance tumour control, albeit requiring rigorous dose adjustment to balance efficacy and safety. Further mechanistic and translational studies have also shown that simultaneous administration of both radionuclides in preclinical prostate cancer models significantly reduced tumour growth, supporting the biological rationale for tandem strategies [65].

Together, these findings suggest that tandem therapy may offer improved tumour control with manageable toxicity, particularly in terms of salivary gland sparing. Nevertheless, current evidence is limited to small pilot series and preclinical data; prospective trials and optimised dosing protocols are required to define the optimal balance between risk and benefit.

## 8. Challenges and Future Perspectives

Despite the remarkable clinical results of [225Ac]Ac-PSMA RLT, several critical challenges remain before widespread implementation in standard oncologic care.

### 8.1. Isotope Availability

A major impediment to the widespread adoption of [225Ac]Ac-PSMA therapy is the global shortage of Ac225. Historically, most of the world’s supply has been derived from the decay of Thorium229 (Th-229), itself a by-product of uranium fuel cycles. However, the annual global production of Ac-225 via this route is estimated to be approximately 68 GBq per year, sufficient for only a few hundred treatment courses, far short of the anticipated clinical demand [15]. Efforts are ongoing to develop accelerator-based production and scale-up of Ac-225 supply, but currently, access to treatment is restricted to specialised centres.

### 8.2. Toxicity Management

Xerostomia remains the principal dose-limiting toxicity. Lack of effective salivary gland protective strategies hinders long-term treatment, especially in patients requiring multiple cycles. Research into gland-sparing approaches (e.g., botulinum toxin, localised cooling, sialoprotective agents) is underway, but robust solutions are still needed.

### 8.3. Lack of Randomised Controlled Trials (RCTs)

As of now, there are no completed prospective RCTs directly comparing [225Ac]Ac-PSMA therapy to standard treatments such as cabazitaxel or [177Lu]Lu-PSMA in mCRPC patients. This gap limits regulatory approval and widespread clinical adoption. However, several ongoing trials are investigating safety, efficacy, dosing, and combination strategies involving [225Ac]Ac-PSMA agents:

❖ AcTION trial (NCT04597411): Phase I dose-escalation study of [225Ac]Ac-PSMA617 in PSMA-positive mCRPC [26].
❖ TATCIST trial (NCT05219500): Phase II study of [225Ac]Ac-PSMAI&T (FPI2265) in patients pre-treated with [177Lu]Lu-PSMA [66].
❖ NCT06402331: Ongoing Phase II/III randomised study of FPI2265 ([225Ac]Ac-PSMA-I&T) in patients previously treated with [177Lu]Lu-PSMA [67].
❖ NCT03276572, NCT04506567, NCT04886986: Studies using antibody-targeted alpha therapy (Ac225 J591), either alone or in tandem with [177Lu]Lu-PSMA or immunotherapy [68].

Although these studies mark significant progress, none have yet reported definitive Phase III results. The field awaits high-quality data to determine the optimal sequencing, comparative benefit, and safety of [225Ac]Ac-PSMA versus existing therapies.

### 8.4. Optimal Sequencing and Combination Strategies

The positioning of [225Ac]Ac-PSMA therapy within the treatment algorithm of mCRPC remains a subject of active investigation. Critical questions under exploration include whether it should be reserved as a salvage option after failure of other treatments, whether it can be effectively combined concurrently with [177Lu]Lu-PSMA (as in tandem approaches), or if earlier-line use might confer enhanced outcomes, particularly in less advanced disease.

Overall, emerging data advocate for a personalised, multimodal strategy, tailoring sequencing and combination regimens of [225Ac]Ac-PSMA with other therapies such as [177Lu]Lu-PSMA, immune checkpoint blockade, poly (ADP-ribose) polymerase (PARP) inhibition, and androgen receptor antagonism, to improve outcomes in mCRPC, a hypothesis that now requires validation in well-designed prospective trials.

### 8.5. Long-Term Safety and Secondary Malignancy Risk

The risk of long-term complications, including t-MDS or secondary malignancies, has not yet been adequately assessed due to insufficient duration of follow-up and the relatively recent adoption of [225Ac]Ac-PSMA therapies. Given the high RBE of alpha-emitters, theoretical concerns persist regarding cumulative DNA damage and mutagenesis in non-target tissues, especially in younger patients or those with prolonged survival. Accordingly, long-term surveillance and registry-based follow-up are strongly recommended moving forward.

## 9. Regulatory and Ethical Considerations

### 9.1. Regulatory Status

To date, [225Ac]Ac-PSMA therapies have not received formal marketing authorisation from leading regulatory bodies such as the European Medicines Agency (EMA) or the U.S. Food and Drug Administration (FDA). As a result, these therapies are used solely under compassionate access programmes, academic or investigator-initiated clinical trials, or through named-patient (expanded access) protocols, reflecting their investigational status.

Recognising the serious unaddressed medical need in late-stage—mCRPC—expedited regulatory pathways such as FDA Fast Track, Breakthrough Therapy Designation, or EMA PRIME may become applicable once robust safety and efficacy data emerge. For instance, a novel compound, Ac-225 FL-020, received FDA Fast Track designation in May 2024, indicating regulatory openness to new Ac-225-based therapies [69].

### 9.2. Ethical Considerations

Equity of access: Ac-225-based treatments are currently limited to specialised centres equipped with dedicated radiochemistry infrastructure and regulated handling facilities. This geographic and institutional limitation, alongside the high cost of radionuclide production and the concentration of manufacturing capacity within a limited number of specialised facilities, contributes to pronounced socioeconomic and geographic disparities in patient access [26,69,70]. Patients in low- and middle-income countries, or those residing far from major nuclear medicine centres, face significant barriers to timely diagnosis, referral, and treatment. These challenges are further exacerbated by the restricted global availability of radionuclides [70]. Such disparities underscore the urgent need for coordinated international initiatives, including shared production facilities, cross-border patient referral frameworks, and harmonised training programmes, to ensure equitable distribution of expertise and resources [26,66,70,71]. Multinational research collaborations, exemplified by ongoing European and global consortia, may provide valuable models for bridging these gaps while accelerating the generation of high-quality evidence [26,66,69].

Informed consent: Given the lack of long-term safety data, especially concerning delayed toxicities such as secondary malignancies or marrow damage, it is ethically imperative that patients receive fully informed consent. This should include explicit discussions about uncertainties surrounding long-term risks, the investigational nature of therapy, and alternative options.

Radioprotection and safety protocols: The use of alpha-emitting radionuclides such as Ac-225 requires stringent radioprotection measures to safeguard healthcare workers and caregivers. This includes secure handling, contamination monitoring (e.g., wipe tests), safe isotope disposal protocols, and adherence to stringent International Atomic Energy Agency (IAEA) guidelines and national radiation safety regulations (IAEA coordinated research project, 2022–2026) [71].

Implementing [225Ac]Ac-PSMA therapy thus demands not only scientific precision but also robust ethical oversight and institutional compliance with radiation protection standards, quality assurance in radiopharmaceutical preparation, and transparent patient communication.

## 10. Conclusions

[225Ac]Ac-PSMA RLT represents a highly promising frontier in the management of mCRPC, particularly in heavily pre-treated patients and those resistant to conventional systemic therapies. With its high LET, short path length, and selective PSMA-targeting mechanism, [225Ac]Ac-PSMA demonstrates potent antitumour activity, especially in micrometastatic and radioresistant disease, while maintaining a manageable toxicity profile under optimised dosing conditions.

Clinical data from both prospective and retrospective studies consistently demonstrate robust biochemical response rates, with subsets of patients achieving long-term remission and prolonged PFS. Notably, recent evidence supports expanding its role earlier in the treatment algorithm, including in chemotherapy-naïve and even hormone-sensitive populations, although further validation is required through high-level evidence.

Toxicity, while generally tolerable, remains a limiting factor. Xerostomia emerges as the most prevalent adverse event and is often dose-limiting, reflecting the physiological expression of PSMA in salivary glands and the local cytotoxicity of alpha particles. Haematologic suppression and renal effects are less frequent but necessitate monitoring, particularly in patients with prior extensive treatment histories. Personalised dosing strategies and tandem regimens with [177Lu]Lu-PSMA are being explored to mitigate toxicity while maintaining therapeutic efficacy.

Despite its clinical potential, several critical barriers impede the routine implementation of [225Ac]Ac-PSMA. These include limited isotope availability, absence of standardised treatment protocols, lack of regulatory approval, and an urgent need for Phase III RCTs to establish comparative effectiveness against current standards. Current access is restricted to named-patient protocols and early-phase trials, although ongoing studies may pave the way for regulatory clearance via expedited pathways such as FDA Fast Track or EMA PRIME.

From an ethical standpoint, the implementation of [225Ac]Ac-PSMA requires careful consideration of equitable access, informed consent, and institutional compliance with radioprotection regulations. The high RBE of alpha particles also raises theoretical concerns regarding long-term mutagenic effects, especially in younger patients or long-term survivors, warranting extended follow-up and registry-based surveillance.

In summary, [225Ac]Ac-PSMA RLT holds substantial promise as a transformative therapy in advanced prostate cancer, with demonstrated activity in difficult-to-treat populations and emerging applications in earlier lines of care. Nevertheless, the path to widespread clinical integration depends on resolving critical challenges related to supply, standardisation, regulatory validation, and ethical oversight. Multinational collaboration, such as that fostered within this European research project, is essential to advance the development, clinical evaluation, and equitable deployment of this novel therapeutic modality.

## Figures and Tables

**Table 1:** Overview of dosing approaches of [225Ac]Ac-PSMA RLT in mCRPC.

References	Study Type	Doses	Administration Protocol	Patients	Treatment Response Based on PSA Value
M. Sathekge et al. (2019) [8]	Prospective	Initial dose 8 MBq; de-escalated to 7, 6, or 4 MBq with good response; one patient escalated to 13 MBq in cycle 3	At 2-month intervals, 14 patients received 3 cycles; treatment stopped after 2 cycles in 3 patients due to good response; 6 patients received additional treatment after cycle 3	17 heavily pre-treated mCRPC patients	≥90% PSA decline in 82% of patients; 41% had undetectable PSA and remained in remission at 12 months; Radiological Response > 50% in 88%
Yadav et al. (2020) [7]	Prospective	100 kBq/kg body weight	2 cycles given 8 weeks apart; at 6-week assessment: stop if disease progresses, continue if stable or responsive	28 mCRPC patients refractory to or without prior [177Lu]Lu-PSMA-617 therapy	50% PSA decline in 25% (initial follow-up) and 39% (end); molecular response in 22/28 patients: 9% complete, 45.4% partial, 9% stable, 36% progressive; disease control: 82% (biochemical), 63.6% (molecular).
D. Y. Lee & Kim. (2022) [33]	Retrospective	1.5–13 MBq/cycle	1–8 cycles	263 patients (9 studies)	Pooled > 50% PSA decline: 61%; any PSA decline: 83.6%.
Kratochwil et al. (2018) [9]	Prospective	100 kBq/kg per cycle	At 8-week intervals, 3–5 cycles per patient	40 mCRPC patients	63% had >50% PSA drop; 87% had any PSA response
M. Sathekge et al. (2020) [34]	Prospective	Initial dose: 8 MBq; reduced to 7, 6, or 4 MBq based on response; given every 8 weeks	Median 3 cycles (range 1–8)	73 mCRPC patients	70% had >50% PSA drop; 82% had any PSA response
Zacherl et al. (2021) [35]	Retrospective	Median/mean dose 7.8 MBq	34 cycles given; most received 1–2 cycles	14 mCRPC patients	50% had >50% PSA drop; 79% had any PSA response
Feuerecker et al. (2021) [36]	Prospective	Median/mean dose 9 MBq (range, 4–13 MBq), every 2 months	Median of 2 (range 1–6)	26 late-stage mCRPC patients	PSA decline of ≥50% was achieved in 17/26 patients
Rosar et al. (2021) [37]	Retrospective	Median/mean of 2.7 ± 1.1 MBq (corresponding to 33 ± 15 kBq/kg)	Median of 2 (range 1–6)	15 mCRPC patients received [225Ac]Ac-PSMA-617 during initial [177Lu]Lu-PSMA-617 RLT	>50% of PSA decline, any PSA decline
Sen et al. (2021) [38]	Retrospective	At 2-month intervals, median/mean of 100 kBq/kg	Median of 2 (range 2–5)	38 mCRPC patients with progressive disease following at least one taxane-based chemotherapy	66% composite response (≥50% PSA decline and/or radiological)
Khreish et al. (2020) [39]	Retrospective	Ac-225: median 5.3 MBq (1.5–7.9) + Lu-177: median 6.9 GBq (5.0–11.6)	1 cycle of tandem therapy; responders continued [177Lu]Lu-PSMA (0–5 cycles)	20 mCRPC patients post-[177Lu]Lu-PSMA-617 monotherapy failure	65% had PSA decline >50%
Satapathy et al. (2020) [28]	Retrospective	Median/mean of 100 kBq/kg/cycle Median cumulative dose: 8.3 MBq	1–4 cycles, at 8–12 week intervals	11 mCRPC patients	46% had ≥50% PSA decline; 27% stable PSA

**Table 2:** Adverse events and their frequencies across studies on [225Ac]Ac-PSMA RLT.

References	Side Effects	Frequency	Patients
Perrone et al. (2025) [52]	Flare pain Grade 3 Anaemia Grade 3 Leukocytopenia Grade 3/4 Thrombocytopenia Grade 3 Nephrotoxicity Grade 3 Hepatotoxicity Xerostomia (G1/G2)	16 patients (22.5%) 15 patients (21.1%) 6 patients (8.4%) 14 patients (19.7%) 1 patient (1.4%) 6 patients (8.4%) 9 patients (12.7%)	71
M. Sathekge et al. (2019) [8]	Grade 1/2 xerostomia Grade 3 anaemia Grade 4 renal toxicity	17 patients (100%) 1 patient (5.9%) 1 patient (5.9%)	17
Ma et al. (2022) [53] Meta-analysis (6 retrospective studies)	Xerostomia (any grade) Xerostomia (grade III) Anaemia (any grade) Anaemia (grade III) Leukopenia (any grade) Leukopenia (grade III) Thrombocytopenia (any grade) Thrombocytopenia (grade III) Nephrotoxicity (grade III) Weight loss Fatigue Anorexia Nausea Constipation Xerophthalmia Hearing loss	155/201 (77.1%) 6/201 (3.0%) 61/201 (30.3%) 15/201 (7.5%) 30/201 (14.9%) 9/201 (4.5%) 30/201 (14.9%) 11/201 (5.5%) 6/201 (3.0%) 54/201 (26.9%) 52/201 (25.9%) 34/201 (16.9%) 26/201 (12.9%) 21/201 (10.4%) 4 cases (2%) 2 cases (1%)	201
Yadav et al. (2020) [7]	Fatigue (grade I/II) Fatigue (grade III) Xerostomia (grade I) Xerostomia (grade II) Xerostomia (any grade) Anaemia (grade III)	14/28 (50%) 1/28 (3.6%) 3/28 (10.7%) 5/28 (17.9%) 8/28 (28.6%) 1/28 (3.6%)	28
D. Y. Lee & Kim. (2022) [33] Meta-analysis of 9 studies	Xerostomia (any grade) Anaemia (grade 3–4) Leukocytopenia (grade 3–4) Thrombocytopenia (grade 3–4)	165/263 (63%) 37/263 (14%) 11/263 (4%) 18/263 (7%)	263
Ling et al. (2022) [54] Meta-analysis (10 studies)	Xerostomia (any grade) Xerostomia (Grade ≥ 3) Xerophthalmia Dysgeusia Fatigue (any grade) Fatigue (Grade ≥ 3) Anorexia Diarrhoea Constipation Nausea Vomiting Anaemia (any grade) Anaemia (Grade ≥ 3) Leukopenia (any grade) Leukopenia (Grade ≥ 3) Thrombocytopenia (any grade) Thrombocytopenia (Grade ≥ 3) Nephrotoxicity (any grade) Nephrotoxicity (Grade ≥ 3)	161/210 (77%) 2/12 (17%) 5/87 (6%) 10/87 (11%) 73/166 (44%) 1/28 (4%) 36/124 (29%) 1/11 (9%) 21/84 (25%) 23/112 (21%) 5/84 (6%) 52/170 (31%) 18/169 (11%) 26/139 (19%) 10/113 (9%) 22/151 (15%) 9/122 (7%) 27/126 (21%) 6/84 (7%)	210 12 87 87 166 28 124 11 84 112 84 170 169 139 113 151 122 126 84
Alan-Selcuk et al. (2023) [55]	Grade 1–3 Haematologic Toxicity Grade 1–3 Nephrotoxicity Xerostomia	4 (17.4%) 1 (4.3%) 23 (100%)	23

**Table 3:** Key physical and radiobiological properties of [225Ac]Ac-PSMA vs. [177Lu]Lu-PSMA.

Property	[225Ac]Ac-PSMA	[177Lu]Lu-PSMA
Radiation type	Alpha (α) particles (4 α particles with energies between 5.8 and 8.4 MeV)	Beta (β) particles
Path length	~47–85 μm	~0.7–2.1 mm
LET	High LET (~100 keV/μm)	Low LET (~0.2 keV/μm)
Physical half-life	~9.9 days	~6.7 days
Cytotoxicity	Highly potent and localised cellular destruction due to short range and high LET	More diffuse cytotoxicity due to extended path and lower LET

**Table 4:** Comparative toxicity profiles of [225Ac]Ac-PSMA and [177Lu]Lu-PSMA RLT.

Toxicity	[225Ac]Ac-PSMA	[177Lu]Lu-PSMA
Xerostomia	Common (70–84%), mainly mild–moderate (G1–2); severe rare (~1–2%)	Occurs in ~8%, generally mild–moderate
Haematologic	Anaemia 10–12%, leukopenia ~6–8%, thrombocytopenia ~5–6%; higher in heavily pre-treated or marrow involved patients	Anaemia ~7%, leukopenia ~3–4%, thrombocytopenia ~4–5%; mostly reversible
Renal toxicity	Rare (~3–4%); isolated deterioration, esp. in patients with baseline renal issues	Uncommon; no grade 3–4 events in some series; mild creatinine elevation seen (~25%)
Long-term effects	Still under evaluation; rare grade 3 toxicities reported (anaemia ~21%, thrombocytopenia ~20%, nephrotoxicity ~1–2%)	Better characterised; toxicities are mostly transient and manageable over time

## Data Availability

No new data were created or analyzed in this study. Data sharing is not applicable to this article.

## Abbreviations

The following abbreviations are used in this manuscript:

TAT	Targeted alpha therapy
mCRPC	metastatic castration-resistant prostate cancer
[225Ac]Ac-PSMA	actinium-225-labelled prostate-specific membrane antigen
LET	linear energy transfer
RLT	radioligand therapy
Lu-177	lutetium-177
PSA	prostate-specific antigen
ADT	androgen deprivation therapy
PFS	progression-free survival
RBE	relative biological effectiveness
ECOG	Eastern Cooperative Oncology Group
PCWG3	Prostate Cancer Clinical Trials Working Group 3
mHSPC	hormone-sensitive metastatic prostate cancer
TV	tumour volume
TL-PSMA	Total lesion PSMA
TLG	total lesion glycolysis
OS	overall survival
Th-229	Thorium-229
RCTs	Randomised Controlled Trials
t-MDS	therapy-related myelodysplastic syndromes
EMA	European Medicines Agency
FDA	Food and Drug Administration
IAEA	International Atomic Energy Agency
PARP	poly (ADP-ribose) polymerase
