# Correction: Jalloul et al. Targeted Alpha Therapy: Exploring the Clinical Insights into [225Ac]Ac-PSMA and Its Relevance Compared with [177Lu]Lu-PSMA in Advanced Prostate Cancer Management. *Pharmaceuticals* 2025, *18*, 1215

**DOI:** 10.3390/ph19030485

**Published:** 2026-03-16

**Authors:** Wael Jalloul, Vlad Ghizdovat, Tamás Pócza, Alexandra Saviuc, Despina Jalloul, Irena Cristina Grierosu, Cipriana Stefanescu

**Affiliations:** 1 Department of Biophysics and Medical Physics-Nuclear Medicine, “Grigore T. Popa” University of Medicine and Pharmacy, 700115 Iasi, Romania; jalloul.wael@umfiasi.ro (W.J.); iuliana.saviuc@gmail.com (A.S.); despina.prisacariu.dp@gmail.com (D.J.); cipriana.stefanescu@umfiasi.ro (C.S.); 2 North East Regional Innovative Cluster for Structural and Molecular Imaging (Imago-Mol), 700115 Iasi, Romania; 3 Institute of Nuclear Techniques, Budapest University of Technology and Economics, H-1111 Budapest, Hungary; poczatamas87@gmail.com; 4 Center of Radiotherapy, National Institute of Oncology, H-1122 Budapest, Hungary

Addition of an Author

Tamás Pócza was not included as an author in the original publication [1]. The authors state that the scientific conclusions are unaffected. This correction was approved by the Academic Editor. The original publication has also been updated.

The affiliation and email of Tamás Pócza appears below:

Institute of Nuclear Techniques, Budapest University of Technology and Economics, H-1111 Budapest, Hungary; poczatamas87@gmail.com

Center of Radiotherapy, National Institute of Oncology, H-1122 Budapest, Hungary

The updated Author Contributions appears below:

**Author Contributions:** Conceptualization, W.J., A.S., V.G. and D.J.; methodology, C.S., I.C.G. and W.J.; software, D.J.; validation, C.S.; formal analysis, I.C.G. and T.P.; investigation, W.J., T.P. and A.S.; resources, W.J. and V.G.; data curation, W.J. and T.P.; writing—original draft preparation, W.J. and A.S.; writing—review and editing, W.J. and D.J.; visualization, V.G.; supervision, I.C.G. and C.S.; project administration, W.J., V.G. and C.S. All authors have read and agreed to the published version of the manuscript.

## References

[1] Jalloul W., Ghizdovat V., Pócza T., Saviuc A., Jalloul D., Grierosu I.C., Stefanescu C. (2025). Targeted Alpha Therapy: Exploring the Clinical Insights into [225Ac]Ac-PSMA and Its Relevance Compared with [177Lu]Lu-PSMA in Advanced Prostate Cancer Management. Pharmaceuticals.

